# Research on Brand Image Evaluation Method Based on Consumer Sentiment Analysis

**DOI:** 10.1155/2022/2647515

**Published:** 2022-05-27

**Authors:** ZhengMin Li

**Affiliations:** School of Journalism & Communication, Wuhan University, Wuhan 430072, Hubei, China

## Abstract

Brand image assessment is a key step to reasonably quantify the value of a brand and has far-reaching significance for improving the competitiveness of an enterprise. With the rapid development of Internet technology, traditional questionnaires can no longer meet the current needs of brand image assessment. In this environment, the huge amount of fragmented consumer topic data provides a rich data resource and new research ideas for brand image assessment. Therefore, a brand image assessment method based on consumer sentiment analysis is proposed. First, a topic-based brand image cognitive label extraction method is proposed by setting language rules, aggregation rules, and ranking rules according to the characteristics of online topic data. Then, the fusion of cognitive labels and deep features is performed by fusing the deep features extracted from word vectors. Finally, a supervised learning support vector machine is selected as the sentiment classification model. The experimental results show that based on the obtained important cognitive labels, enterprises are able to better understand the unique attributes that consumers have for the brand; the feature fusion approach is better evaluated and can accurately reflect consumers' views on brand image and quantified as brand score.

## 1. Introduction

With the rapid development of the global market, various brands are becoming more and more colorful, but the trend of product homogeneity is intensifying, leading to increasingly fierce competition among enterprises. For an enterprise to gain lasting vitality, it must have a clear and superior brand image. Brand image assessment is a key step in understanding brand image and is the first step in improving brand quality and developing brand communication strategies. Enterprises need a way to objectively assess their brand image. This will enable companies to better grasp consumers' psychological perceptions and thus develop precise marketing strategies that vary from person to person and can provide support and more insight into future decisions. Internet technology has broken the traditional ecology of information dissemination and changed the previous way of information delivery. The traditional brand evaluation method based on a questionnaire survey can no longer meet the current demand for brand image evaluation.

In the new information communication ecology, user-generated content brings unprecedented opportunities and challenges for brand image mining [1–3]. Based on user-generated data, social networks can improve the feasibility, operability, and flexibility of brand image assessment. Analyzing the sentiment tendency of texts through natural language processing techniques for user-topic texts is a new trend in brand image assessment and has a very practical application value [4–8]. The task process of sentiment analysis includes sentiment feature extraction and sentiment classification. The first task of sentiment feature extraction is to find the words (features) that represent the user's point of view and then classify the features for sentiment tendency [9–11]. From a psychological perspective, the brand image refers to the sum of consumers' psychological feelings about the brand's products and services. Brand image is divided into two main dimensions, one that is the user's perception of the functional attributes of the brand and one that reflects the emotional value of the brand.

Consumer perception of a brand is the overall impression of the consumer of the brand [12]. The most basic element of brand image assessment is brand perception. The explosive growth of topic texts provides a massive source of data for brand perception analysis. Therefore, this paper focuses on brand research through topic text sentiment analysis techniques, so as to extract the attributes of brands that consumers pay attention to. It also quantifies consumers' attitudes toward brands into brand scores, so that companies can understand consumers' views more intuitively and objectively, understand the strengths and weaknesses of brand images, and provide them with decision support for resource allocation and product strategy adjustment.

The rest of the paper is organized as follows: In Section 2, the related research is studied in detail, while Section 3 provides the detailed methodology of brand image cognitive label extraction.  Section 4 provides the detailed brand image evaluation method based on a weighted fusion model.  Section 5 provides the results and discussion. Finally, the paper is concluded in Section 6.

## 2. Related Research

Brand image theory was first proposed in the United States in the 1960s. After that, brand image theory has been developed continuously, and brand image is gradually interpreted as a collection of consumers' perception of various elements and concepts of a brand. Brand image in general can be divided into two categories, one from the image visual perspective and one from the psychological perspective.

The visual image of a brand refers to the visual design and communication elements that are visible to the user. As the differences between products become less and less, brands are anthropomorphized. Rehman et al. [13] mapped consumers' personality traits vaguely to the brand image. Consumers and brands are closely linked, and consumption can interact and communicate through brands. Portal et al. [14] described brands anthropomorphically and considered brands as having their own independent personalities. The relationship between the consumer and the product is also an interpersonal relationship. These early definitions of anthropomorphism were vague until the concept of the brand image became clearer by using the Big Five personality theory to construct a brand personality theory.

From a psychological perspective, the brand image refers to the sum of consumers' psychological feelings about a brand. Psychological image is divided into two main dimensions, one that is the user's perception of the functional attributes of the brand and one that reflects the emotional value of the brand. The perception of functional attributes refers to the ability of the brand to satisfy functional needs [15]. For example, a car can fulfill the function of a substitute, and coffee can refresh the mind. Currently, defining brand image at the psychological level has been dominant in the field of related research. Sentiment analysis from a psychological perspective consists of two components: feature selection of texts and feature-based sentiment classification models. Early sentiment analysis was mainly based on a rule-based feature approach to classification. Recently, many researchers have started to study machine learning classification for sentiment analysis methods. Suhasini et al. [16] used a fusion of machine learning and rules for sentiment classification of microblog data with good results and solved the problem of sentiment classification of fuzzy words by combining rules and statistics. Kang et al. [17] compared plain Bayes, support vector machine (SVM), and Rocchio (ROC) classification models, where the second classifier effect is the best performance among the three. The above findings suggest that machine learning-based classification methods are more scalable, while rule-based classification methods need to be constrained by the corpus.

In this paper, we analyze massive topic sentiment data for brand evaluation and set language rules, aggregation rules, and ranking rules to extract consumers' cognitive label (CL) of the brand. This cognitive label is used to achieve a shallow portrait of the brand. Meanwhile, the feature selection method incorporating deep features improves the evaluation effect of the classification model. Finally, a supervised learning support vector machine is selected as the sentiment classification model.

The main innovation points and contributions of this paper are as follows.

1. A topic sentiment-based brand image cognitive analysis method is proposed to extract the features of topic text and improve the effect of the sentiment recognition algorithm. By regularizing the topic data (constraint rules), consumers' cognitive labels of brands are extracted.
2. In order to make up for the defect that shallow features cannot obtain complete semantic information, this paper introduces the method of fusing deep features, fusing shallow learning features with deep learning features to solve the problem of recognizing a large number of nonentity words in the text, enriching the set of features used by the classification algorithm, and improving the correct rate of text classification.

## 3. Brand Image Cognitive Label Extraction Based on Topic Sentiment

The effect of sentiment classification is crucial with feature selection, and this paper proposes a rule-based cognitive feature extraction method and aggregates cognitive labels with the help of a synonym dictionary and Jaccard similarity. Finally, the TF-MF model is applied to calculate the importance of cognitive labels.

### 3.1. Language Rules for Cognitive Label Extraction

Definition of cognitive label: users' knowledge and understanding of brand connotation. For example, the Redmi Note, which positions itself in the low and midrange market, users' cognitive labels of its brand are “within one thousand dollars,” “teenager,” “big screen,” “good pixel,” “lagging,” etc. In this paper, *w* is used to denote the cognitive labels.

Let the corpus set *T*={*t*_1_, *t*_2_,…, *t*_*n*_}, where *t*_*i*_ denotes a corpus instance. Different users use different lengths of utterances to express their feelings or experiences about a brand. In general, a single phrase may contain several aspects of a user's perception of a brand. However, one aspect of the user's perception of a brand is usually not in several phrases, but in a single phrase. For example, “It's really good! The system is smooth, the screen is good, the performance is good, but unfortunately there is no transparent case. [Big Love] It's really good!.” In this corpus, the idea of “no transparent case” can only be expressed in one phrase. However, the phrase “smooth system, good screen, and good performance” contains the perceptions of “smooth system,” “good screen,” and “good performance.” The corpus is partitioned using the dot mark set $D=\left\{\begin{array}{ccccc} , & ; & : & ? & ! \end{array}\right\}$ to obtain a set *S*={*s*_1_, *s*_2_,…, *s*_*m*_} of phrases, where *m* > *n*. We choose phrase *s*_*i*_ as the basic unit to extract the candidate cognitive labels.

After splitting the input text into a series of phrase collections *S*, the traditional feature extraction view is to deal with adjectives. However, in addition to adjectives, nouns, adverbs, and verbs are often used to express opinions and perceptions. For example, “big” as an adjective is often used to modify nouns. If “big brand” is the user's perception of the brand, then this noun phrase can be regarded as the user's perception label. Similarly, verbal and adverbial phrases such as “stuck” and “trustworthy” are also user awareness, reflecting the user's perception of a particular aspect of the brand. After splitting and deactivating *s*_*i*_, the speech chain rule set *R*={*r*_1_, *r*_2_, *r*_3_, *r*_4_, *r*_5_, *r*_6_} is used to extract candidate cognitive labels *w*, as shown in Table 1.

**Figure 1:**
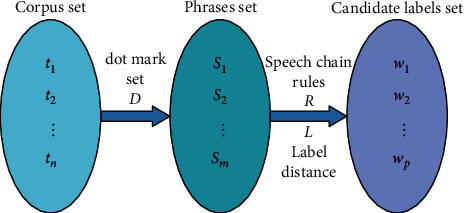
Sketch map of candidate labels extracting based on language rules.

The distance between the first word and the last word in the label is *L*. There are too many interferences in the labels extracted by the lexical chain rule, so we need to use the distance principle to filter the interferences. In the corpus “The phone is very comfortable to operate,” after preprocessing, the corpus becomes “phone/n, operate/vn, very/d, comfortable/a.” We may extract the label “phone comfortable.” At this point, “phone” is in position 1, and “comfortable” is in position 4. Using the principle of closest modification, we design a word spacing constraint. The label extraction is constrained by using the label distance *L* to improve the readability of the cognitive labels. After the above steps, a series of candidate cognitive tag sets *W*={*w*_1_, *w*_2_,…, *w*_*p*_} are obtained from the corpus *T*. The processing schematic is shown in Figure 1.

### 3.2. Aggregation Strategies for Cognitive Label Extraction

The initial extracted cognitive labels appear to be messy and voluminous because users freely choose their own terms when expressing their opinions and views. Therefore, we need to further aggregate the initial cognitive labels extracted in the previous section. Although users are free to use words when expressing their perceptions, in most cases, users' perceptions of a certain side are often near or opposite words. Therefore, we use Jaccard similarity [18–20] to cluster the extracted tags based on a lexicon of near-sense words. For the candidate tags *w*_*i*_ and *w*_*j*_ of the same length, the similarity between them is

(1) $$\begin{array}{c} \mathrm{SIM}\left(w_{i},w_{j}\right)=\frac{\left|w_{i}\cap w_{j}\right|}{\left|w_{i}\cup w_{j}\right|}. \end{array}$$

When a pair of elements in a phrase appears in pairs in the list of near-synonyms, we consider them to be the same word. For example, *A* = “Beautiful appearance,” *B* = “Pretty appearance,” *C* = “Fast logistics.” Obviously, *A* and *B* have a high similarity. The words “Beautiful” and “Pretty” are a set of near-synonyms, so SIM(*A*, *B*) = 1 and SIM(*A*, *C*) = 1/5 = 0.2.

**Figure 2:**
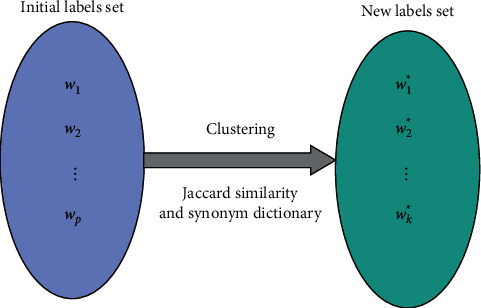
Candidate labels aggregation diagram.

Based on the Jaccard similarity and synonym dictionary, we can get the similarity between any two words, which in turn clusters the initial cognitive labels. The word *w* with the highest frequency in this cluster is selected as the representative of this cluster. At this point, the new set of cognitive labels *W*^*∗*^={*x*_1_^*∗*^, *x*_2_^*∗*^,…, *x*^*∗*^, }, *k* ≤ *p* is obtained, as shown in Figure 2. For example, after the aggregation of “good endurance, great endurance, OK endurance,” the cognitive label “good endurance” is extracted.

### 3.3. Importance Ranking of Cognitive Labels

In order to understand the importance of the acquired cognitive labels in the minds of consumers, the importance of the cognitive labels needs to be ranked. The entire corpus set is *T,* and the set of tags appearing in this corpus is *w*^*∗*^. The elements in the initial tag set *W* are replaced by representative elements in the same tag cluster. *f*_*w*^*∗*^_ denotes the number of occurrences of the word *w*^*∗*^ in the corpus set *T*, and *B* denotes the number of corpus containing the word *w*^*∗*^ in the corpus set. Considering that *w*^*∗*^ is already a label with actual meaning, the importance of the label *w*^*∗*^ can be calculated according to the TF-MF model.

(2) $$\begin{array}{c} \mathrm{TFMF}\left(w^{*}\right)=\frac{f_{w^{*}}}{\sum_{W^{*}}f_{w^{*}}}\times \;\log\left(\frac{B}{\left|T\right|}+1\right). \end{array}$$

**Figure 3:**
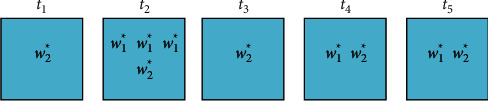
Example of TFMF model.

An example of the TF-MF model is given as follows. Suppose the corpus set *T* has five different corpora *t*_1_, *t*_2_, *t*_3_, *t*_4_, *t*_5_. Two cognitive labels *w*_1_^*∗*^ and *w*_2_^*∗*^ appear in the corpus, and the specific distribution is shown in Figure 3.

The importance of the two was calculated by the TF-MF model.

(3) $$\begin{array}{c} \mathrm{TFMF}\left(w_{1}^{*}\right)=\frac{5}{10}\times \;\log\left(\frac{3}{5}+1\right)=0.102,\\ \mathrm{TFMF}\left(w_{2}^{*}\right)=\frac{5}{10}\times \;\log\left(\frac{5}{5}+1\right)=0.151. \end{array}$$

Although the two labels *w*_1_^*∗*^ and *w*_2_^*∗*^ appear equally in the corpus in total, the label *w*_2_^*∗*^ is more important than *w*_1_^*∗*^. This coincides with the fact that in practice the more people pay attention to it the more it deserves attention.

## 4. Brand Image Assessment Method Based on Weighted Fusion Model

The previous section proposed to extract features by cognitive labels, but cognitive labels only address the importance of brand attribute features and cannot explain the meaning of a large number of noncognitive labels. To complement this deficiency, this paper then introduces fusion features to supplement the feature vectors based on the results of deep learning word vector models.

### 4.1. Features Based on Word Vector Model

Since the implicit meaning of some words can be obtained using deep model parameter passing, this paper uses the CBOW (continuous bag of words) model [21] to train the word vector model. The CBOW model uses the context before and after the target word to predict the probability of occurrence of the target word. We used a three-layer word vector model for training to obtain the word vector representation.

Firstly, a lexicon matching and hidden Markov model-based lexicographer are used to split the text. Then, the lexicon numbers are extracted from the split text, and the numbers are used to convert the training text utterances into id representations. Finally, the word2vec tool [22] is called, and the word vector model is trained according to the belonging parameter settings. The dimensionality of the word vector is set to 200, and the sliding window size in the training model is 5. The output results in a vector set of floating-point numbers and the vector values are normalized.

### 4.2. Emotion Classification Model with Feature Fusion

We fuse shallow cognitive label extraction and deep feature filtering to improve the accuracy of feature selection and optimize the classification results. Then, the training text is trained for classification using an SVM classifier, which we call CL-C-SVM.

**Figure 4:**
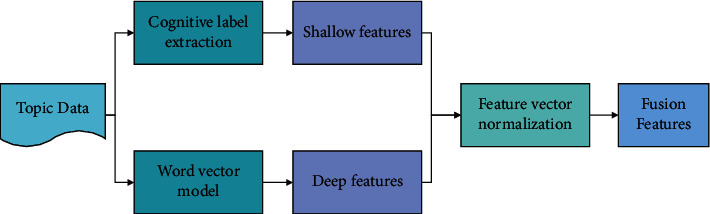
The process of feature fusion.

The processing of text content in sentiment analysis can be reduced to the processing of a vector of a certain length. For the extracted cognitive labels, they can be represented in the form of one-hot. Each cognitive label represents a dimension, and a sentence may be represented by thousands of dimensions. The position of words that have appeared in the sentence is filled with 1, and the position corresponding to words that have not appeared is filled with 0. This allows the text data to be represented by a high dimensional 0-1 vectorization. The extracted cognitive labels and the word vectors from the training output are fused to generate new feature files, and the flow of feature fusion is shown in Figure 4.

### 4.3. Definition of Brand Image

For a brand, which contains a lot of products, products at the same time contain a lot of models, different models of goods in the e-commerce site, and a large number of sellers, thus generating a large number of topic comments data. The good or bad topic comments can represent the brand's reputation to a certain extent. According to the principle of statistics, in the context of big data statistics, good or bad user word-of-mouth can be used as a standard for a high or low brand image, so the brand assessment given in this paper is defined as

(4) $$\begin{array}{c} \text{score}=\sum_{i=0}^{m}w_{ij}-\sum_{i=0}^{n}w_{ij}, \end{array}$$

where *m* denotes the number of positive comments and *n* denotes the number of negative comments. *w*_*ij*_ denotes the ranking of each cognitive label *j* in the sentence *i*.

## 5. Experiment and Result Analysis

### 5.1. Data Selection and Preprocessing

**Figure 5:**
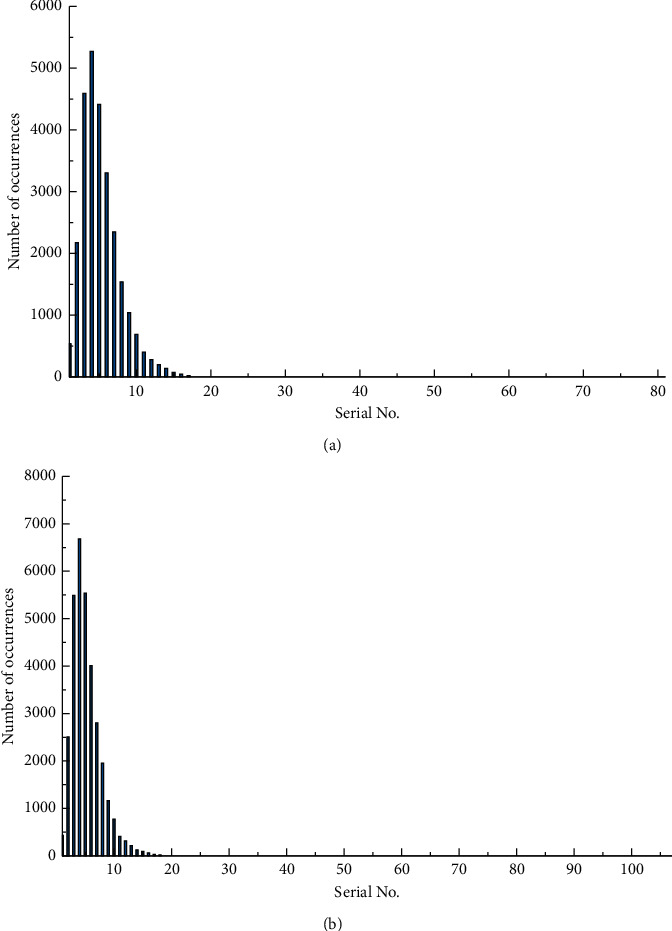
Short sentences' length distribution. (a) Huawei Mate 50. (b) Samsung Galaxy S22.

Two competing cell phone brands, <Huawei mate50> and <Samsung Galaxy S22>, were selected for the experiment. Records about these two brands were searched on three online platforms, namely B2C e-commerce cell phone category comment information, Sina Weibo and Zhongguancun Online, and 10,000 records each were randomly selected as the experimental data set. Each corpus was segmented with the dot as the separator to obtain the basic unit phrases for label extraction. The NLPIR system was used for word segmentation and lexical annotation, and the deactivated words in the records were removed using a deactivated word list. The Huawei mate50 dataset has 27575 phrases, with a maximum of 163 words and a minimum of 1 word in the phrase, and an average length of 5 words. The Samsung Galaxy S22 dataset has 34563 phrases, with a maximum of 195 words and a minimum of 1 word, and each phrase has an average of 5 words. The overall distribution is shown in Figure 5.

### 5.2. Cognitive Labeling Analysis

Using the speech chain rule, the initial set of cognitive labels is obtained, and the results are shown in Table 2.

The above table shows that the initial cognitive labels extracted at this point are cluttered and disorganized. Next, critical information needs to be obtained through linguistic aggregation and importance ranking methods. Based on Jaccard similarity and synonym dictionary to calculate the similarity between tags, the initial cognitive tags are aggregated, the tags are ranked using TF-MF rules, and the top 5 tags are taken, as shown in Table 3.

From Table 3, it can be seen that the method in this paper can effectively extract the cognitive labels of consumers, so as to obtain the key elements of users' perception of brand image. Through the method in this paper, it can help companies recognize their image in consumers' mind and discover the most and least satisfied areas of users, thus providing strong support for subsequent brand image evaluation.

### 5.3. Classification Performance

**Figure 6:**
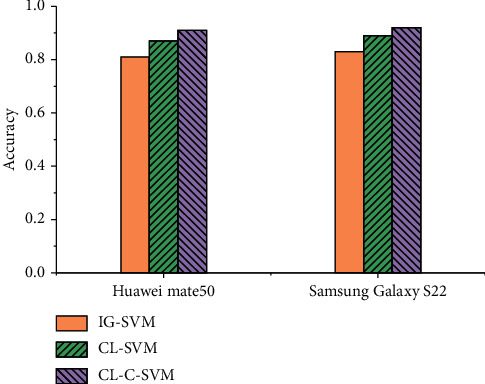
Accuracy of different classification models.

The dataset was annotated manually and labeled with the review data sentiment classification as positive or negative reviews. The labeled data were divided into a training set and a test set in the ratio of 9 : 1. The classical IG (information gain)-SVM model was selected as the comparison algorithm to compare with the proposed CL-SVM and CL-C-SVM classification algorithms to verify their effectiveness. The metrics for evaluating the algorithms were chosen as accuracy rates commonly used in the field of natural language. The accuracy comparison of the different classification models is shown in Figure 6.

From the accuracy comparison shown in Figure 6, it is clear that the CL-C-SVM algorithm proposed in this paper has the best sentiment classification effect. By analyzing the data, the reason can be obtained that the choice of IG, CL, or feature fusion by the feature extraction algorithm leads to a large difference in the actual results. The fused features are more comprehensive and compensate the problem that shallow features cannot explain the meaning of a large number of non-CL words. Combining the shallow filtered CL with deeper features that contain more hidden meanings can improve the accuracy of feature selection.

### 5.4. Brand Image Evaluation Experiment

For the two cell phone brands, the results of brand image assessment using the CL-C-SVM model are shown in Table 4.

**Figure 7:**
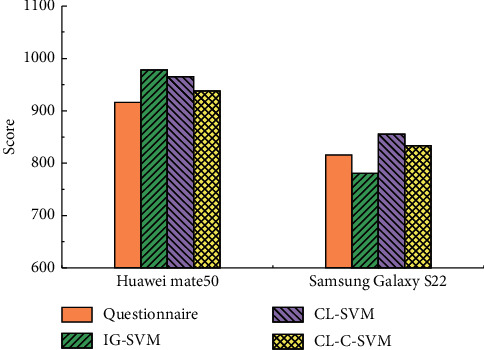
Comparison of brand scores.

In order to conduct a manual and subjective assessment of brand image ratings, this paper adopted a user questionnaire + ranking approach. For the above two cell phone brands, 1000 online questionnaires were randomly distributed at the entrance of a large supermarket. The target audience was consumers who had used these two cell phone brands, and they were asked to rate these two cell phone brands. A score of 1 was set as a passing score, and the higher the score, the better the brand's psychological impression on users. The scores and the number of people were multiplied to obtain the average score and normalized to within 1000, and the final results were obtained as shown in Table 5. The comparison of brand scores for different methods is shown in Figure 7.

The scores of different brands are shown in Figure 7, and it is found that the brand scores calculated by the CL-C-SVM model have the best fit with the scores of the user questionnaire. The brand scores derived from the CL-SVM model are slightly less effective but better than those calculated by the IG-SVM model. The practical value of the CL-C-SVM model was verified by comparing the results with those of the manual questionnaire.

## 6. Conclusions

In this paper, a brand image cognitive analysis method based on topic sentiment is proposed to extract features of topic text and improve the effect of the sentiment recognition algorithm. At the same time, the feature selection method incorporating deep features makes up for the defect that shallow features cannot obtain complete semantic information and enriches the set of features used by the classification algorithm. The experimental results show that the proposed cognitive label extraction method can effectively obtain the key elements of users' brand image and discover the most and least satisfied areas of users. In addition, the CL-C-SVM model can accurately quantify consumers' brand scores on brand image, and the results are almost consistent with the manual questionnaire approach. However, the extraction of cognitive labels does not take into account the influence of unexpected events, resulting in the assessment of the model failing to reflect the real-time changes in corporate image, which is important in practical applications. Therefore, further work on this issue will be conducted in subsequent studies.

## Figures and Tables

**Table 1:** Speech chain rules in label extraction.

Rules	Speech chain	Example
*r*_1_	n + d + a (noun + adverb + adjective)	Cost very effective
*r*_2_	n + h + a (noun + prefix + adjective)	Logistics super-fast
*r*_3_	a + n (adjective + noun)	Smooth system
*r*_4_	d + v (adverb + verb)	Very much like
*r*_5_	d + n (adverb + noun)	Very youth
*r*_6_	d + a (adverb + adjective)	Really good

**Table 2:** The distribution of candidate cognitive label in the speech chain.

Candidate label type	n + d + a	n + h + a	n + a	d + a	d + v	d + n	Total
Huawei Mate 50	2954	27	1171	587	3124	2358	10221

Samsung Galaxy S22	4067	55	1466	794	3458	2783	12623

**Table 3:** Cognitive label extraction results.

Cognitive label of Huawei Mate 50	Cognitive label of Samsung Galaxy S22
Fast speed	Great service
Very satisfied	Nice phone
Cost very effective	Nice screen
Battery not durable	Beautiful appearance
Nice screen	Battery not durable

**Table 4:** Score of brand image evaluation.

Candidate label type	Good review	Poor reviews	Medium rating	Normalized mean score
Huawei Mate 50	14112	8427	5036	938.3

Samsung Galaxy S22	16036	11402	7125	833.4

**Table 5:** Scoring results of the questionnaire.

Candidate label type	1 score	2 score	3 score	4 score	5 score	Normalized mean score
Huawei Mate 50	65	82	256	366	241	916.5

Samsung Galaxy S22	73	147	386	231	163	816

## Data Availability

The raw data supporting the conclusions of this article will be made available by the authors, without undue reservation.
